# Human Metapneumovirus in Severe Respiratory Syncytial Virus Bronchiolitis

**DOI:** 10.3201/eid0903.020289

**Published:** 2003-03

**Authors:** Julie Greensill, Paul S. McNamara, Winifred Dove, Brian Flanagan, Rosalind L. Smyth, C. Anthony Hart

**Affiliations:** * University of Liverpool, Liverpool, United Kingdom

**Keywords:** Human metapneumovirus,respiratory syncytial virus, severe bronchiolitis, RT-PCR, dispatch

## Abstract

Reverse transcription-polymerase chain reaction was used to detect segments of the M (matrix), N (nucleoprotein), and F (fusion) genes of human metapneumovirus in bronchoalveolar fluid from 30 infants with severe respiratory syncytial virus bronchiolitis. Seventy percent of them were coinfected with metapneumovirus. Such coinfection might be a factor influencing the severity of bronchiolitis.

Bronchiolitis is an important cause of illness and death in infants. An estimated 25 in every 1,000 children will require hospitalization with bronchiolitis; for 1% to 2% of these children, infection will be severe enough to require ventilatory support (*1*). The etiologic agents of bronchiolitis include adenovirus, influenza and parainfluenza viruses, and *Bordetella pertussis.* However, most infections are due to respiratory syncytial virus (RSV) (*2*). In June 2001, Osterhaus and colleagues reported the discovery of a “new” human respiratory virus (*Pneumovirinae* subfamily, *Paramyxoviridae* family) (*3*). RSV is in the genus *Pneumovirus,* and the new virus, although related to RSV, is more closely related to avian pneumovirus serotype C (previously known as turkey rhinotracheitis virus) (*4*), the only other member of the *Metapneumovirus* genus. The new virus, however, does not infect chickens or turkeys (*3*), and they are unlikely to be a zoonotic source. Thus, the new virus was termed human metapneumovirus (hMPV). Of 28 isolates, all isolated in the winter, 13 came from infants <12 months of age who had disease patterns resembling those seen in RSV infections. Subsequently, human metapneumovirus has been detected in children in Australia (*5*) and Canada (*6*).

## The Study

As part of a larger study examining the immunopathogenesis of severe bronchiolitis, nonbronchoscopic bronchoalveolar lavage samples were collected from 30 infants ventilated with RSV bronchiolitis (determined by antigen detection in nasopharyngeal aspirates) in 2000 and 2001. The sampling was performed according to recent European Respiratory Society guidelines (*7*). Briefly, a suction catheter was passed down the endotracheal tube beyond the bifurcation of the bronchi. Two 1-mL/kg aliquots of sterile normal saline were instilled separately down the suction catheter. Lavage fluid was recovered with constant suction pressure into a mucous trap. Samples were frozen at –80°C until analyzed. Control samples were obtained in winter from children within the same age range who were being intubated and ventilated for noninfection-related conditions. The local research ethics committee approved the study, and informed consent was obtained from parents.

Detection of the human metapneumovirus genome was performed by reverse transcription-polymerase chain amplification (RT-PCR) of the matrix (M), fusion (F), and nucleoprotein (N) genes. RNA was extracted from the specimens by the guanidine/silica method (*8*), and 15 µL was added to a 50-µL RT reaction with a polyT primer and 1.25 U AMV-RT (Gibco, Basingstoke, U.K.) to produce cDNA. Each PCR reaction contained 10 µL of cDNA with 2.5 U Amplitaq Gold (Applied Biosystems, Warrington, U.K.), 2.0 mM Mg Cl_2_, 300 µM deoxynucleoside triphosphate, 0.4 µM of each primer, and the buffer supplied. For amplification of the M gene, primers hMPV-MF1 (AAG TGA ATG CAT CAG CCC AAG) and hMPV-MR1 (CAC AGA CTG TGA GTT TGT CAA A), which amplified the region between nucleotides 212 and 331, were used to give an amplicon of 120 bp. PCR cycling conditions were 95°C for 10 min followed by 45 cycles of 95°C for 1 min, 58°C for 1 min, 72°C for 1 min, and a final extension step of 72°C for 10 min in a Perkin-Elmer (Warrington, Cheshire, U.K.) 2400 model thermal cycler. Positive samples were confirmed by PCR amplification for the F or N gene products, or by amplification of a longer product of the M gene. The longer M product used primer hMPV-MR1 and a new primer, hMPV-MF2 (ATG GAG TCC TAT CTA GTA GAC A), amplifying the region between nucleotides 1 and 331 of the M gene, yielded a 331-bp product. For the F PCR, primers hMPV-FF1 (GTG AGC TGT TCC ATT GGC AG) and hMPV-FR1 (CCC TCA ACT TTG CTT AGC TGA TA) amplified the region between nucleotides 1162 and 1295 of the F protein to give a 134-bp PCR product. PCR cycling conditions were as for the M protein but with an annealing temperature of 62°C. The N PCR used primers hMPV-NF1 (GTA TTA CAG AAG TTT GTT CAT TGA G) and hMPV-NR1 (GAG AAC AAC ACT TGC AAA GTT GG), which amplified the region between nucleotides 710 and 1034 of the N protein to give a 325-bp product. PCR cycling conditions were as for the F protein. In each case, the primers were designed by aligning the M, N, and F gene sequences of the original Dutch isolates (93.1, 93.2, 93.3, 94.1, 94.2, 99.1, 99.2, and 00.1) (*3*) to define regions that were held in common. The primers did not amplify sequences from either human RSV or avian pneumovirus.

Selected PCR products were cloned into a TA cloning vector (pGEM-T, Promega, Southampton, Hampshire, U.K.), and the sequence was determined to confirm the identity of the virus detected by the PCR reaction. Sequences from the M, N, and F proteins of one virus (LIV08) have been deposited in EMBL, with the accession numbers AJ439505, AJ439504, and AJ439503, respectively.

The phylogenetic relationship between isolates was examined. Human metapneumovirus sequences were aligned with the CLUSTALX multiple alignment program (EMBL, Heidelberg, Germany), and analyses were carried out by using the Phylogeny Inference Package (PHYLIP version 3.5c, (J. Felenstein and the University of Washington). DNA sequences were analyzed by using DNADIST followed by neighbor joining; 100 replica samplings were analyzed. Detection of RSV by RT-PCR and N gene typing was as described (*9*).

M gene and at least one other of N, F, or long M amplicons of human metapneumovirus were detected in bronchoscopic bronchoalveolar lavage fluids from 21 (70%) of 30 infants ventilated for RSV bronchiolitis (Table). Four of the M-gene amplicons were cloned and sequenced. Amplicons were cut from agarose gels, eluted, and purified by using a gel purification kit (QIAGEN, West Sussex, U.K.). The amplicons were cloned into pGEM-T (Promega), and sequencing was conducted with MI3 primers by Lark Technologies (Essex, U.K.). In each case, the amplicons were very similar (1 base different) to each other and closely related to those reported for human metapneumovirus (82% to 97%) (*3*). The Figure shows a neighbor-joining tree that uses a 102-bp (nucleotide 208–309) region of the M gene, demonstrating the relationship of a Liverpool (LIV08) strain to those from Holland. RSV was detectable by RT-PCR in only 24 (80%) of 30 nonbronchoscopic bronchoalveolar lavage samples, despite RSV antigen’s being detectable from all nasopharyngeal aspirates at the onset of illness. Of these 24, 23 could be assigned an N genotype (18 NP-4, 4 NP-2, 1 NP-3). Human metapneumovirus coinfection was detectable with each of the genotypes, although only one (25%) of the four infants infected with NP-2 was coinfected with human metapneumovirus, compared with 13 (76%) of 17 of those with NP-4. Human metapneumovirus was detectable in three infants from whom no RSV amplicon could be obtained. Neither RSV nor human metapneumovirus amplicons were detectable in nonbronchoscopic bronchoalveolar lavage fluid from 10 control patients ventilated for reasons unrelated to bronchiolitis. Most infants have serologic evidence of infection with RSV by 2 years of age (*10*), but other than host factors such as underlying cardiac, respiratory, or genetic factors and prematurity, why severe disease develops in some infants and mild disease in others is unclear. For example, although 87% of children have antibodies to RSV by age 18 months (*11*), most children have only upper respiratory tract symptoms such as rhinitis, cough, and coryza (*12*). The age of the patient is also important because lower respiratory tract signs develop in up to 40% of infants <1 year, and 0.5% to 2% of all infants need to be hospitalized (*12*). Coinfection with RSV and other pathogens has been described previously. In studies in Northern Ireland (*13*) and India (*14*), coinfection occurred in 1.1% (all with rhinovirus) and 15% (with influenza and parainfluenza viruses) of patients with RSV acute lower respiratory tract infection, respectively. However, apart from in viral infections that might alter host immunity (*15*), acute respiratory tract infections involving RSV alone were not significantly different from those caused by RSV and another virus (*16*). RSV and human metapneumovirus might have similar seasonal patterns (*3*), so coinfection is possible. In the present study, we detected coinfection with human metapneumovirus in 70% of infants with RSV bronchiolitis sufficiently severe to require admission to the pediatric intensive-care unit for ventilatory support.

**Table:** Characteristics of infants with severe bronchiolitis^a^

Pt. no.	Age (weeks)	Weight (kg)	Predisposing factors		RSV		hMPV		Days of illness before BAL		
1	3	3.5		None		NP-4		+		8	
2^b^	48	6.6		Premature, BPD		–		+		6	
3	18	7.5		None		NP-4		+		7	
4	35	6.0		Cardiac, genetic		–		+		7	
5	11	2.8		Premature		NP-4		+		6	
6	21	5.5		Premature		NP-4		+		4	
7	3	2.3		Premature		–		–		7	
8	5	2.5		Premature		NP-4		+		3	
9	18	5.1		Premature, cardiac		NP-2		–		6	
10	11	5.7		None		NP-4		+		10	
11	21	7.5		None		NP-2		+		9	
12	9	2.0		Premature		NP-4		–		3	
13^b^	16	4.4		Premature, cardiac		NP-2		–		3	
14	7	2.6		Premature		NP-3		+		5	
15	15	2.8		Premature		NP-4		+		5	
16	4	4.0		None		NP-4		+		2	
17	7	4.3		None		NP-4		+		5	
18	12	3.2		Premature, cardiac		NP-4		–		6	
19	12	5.4		None		–		+		13	
20	5	3.2		None		NP-4		+		7	
21	5	2.8		Premature		NP-2		–		5	
22	9	4.7		Cardiac		NP-4		–		8	
23^b^	22	5.7		Genetic		NP-4		+		4	
24	7	3.4		Premature		+		+		11	
25	19	4.8		Premature, BPD		NP-4		–		6	
26	13	3.4		Premature		NP-4		+		6	
27	8	2.5		Premature		NP-4		+		6	
28	29	3.5		Premature, BPD		–		+		6	
29	3	4.1		None		NP-4		–		5	
30	5	4.1		None		–		+		5	

^a^Abbreviations used: Pt., patient; RSV, respiratory syncytial virus; hMPV, human metapneumovirus; BPD: bronchopulmonary dysplasia; BAL, bronchoalveolar lavage; NP, nucleoprotein genotype. ^b^Died.

**Figure:**
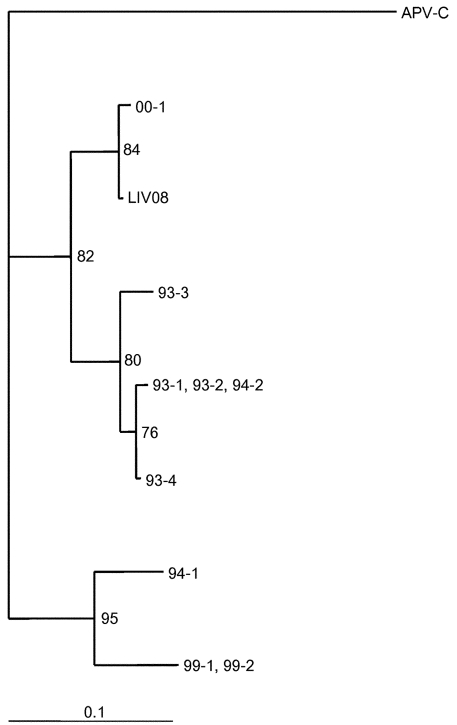
The phylogenetic relationship of human metapneumovirus from Liverpool (LIV08) to those from Holland and to avian pneumovirus. The divergence bar is shown at the bottom of the figure.

Sequence analysis of a portion of the M gene from four of the detected human metapneumoviruses indicated that they were closely related both to each other and to the Dutch isolates. This high rate of coinfection raises the possibility that coinfection with RSV and human metapneumovirus might be another determinant of RSV disease severity. Indeed, of 10 studied infants with severe RSV bronchiolitis and no other risk factors for severe disease, 9 (90%) were coinfected with human metapneumovirus. Confirmation of a role for coinfection will require longitudinal studies over several bronchiolitis seasons, comparing disease severity with frequency of coinfection across the full spectrum of RSV disease. Finally, this high RSV–human metapneumovirus coinfection rate in severe disease means that previous studies into RSV subtype/genotype, disease severity, and cytokine and chemokine expression in RSV disease may need to be reevaluated.
